# Phytate Degradation, Transcellular Mineral Transporters, and Mineral Utilization by Two Strains of Laying Hens as Affected by Dietary Phosphorus and Calcium

**DOI:** 10.3390/ani10101736

**Published:** 2020-09-24

**Authors:** Vera Sommerfeld, Adewunmi Omolade Omotoso, Michael Oster, Henry Reyer, Amélia Camarinha-Silva, Martin Hasselmann, Korinna Huber, Siriluck Ponsuksili, Jana Seifert, Volker Stefanski, Klaus Wimmers, Markus Rodehutscord

**Affiliations:** 1 Institute of Animal Science, University of Hohenheim, 70599 Stuttgart, Germany; v.sommerfeld@uni-hohenheim.de (V.S.); amelia.silva@uni-hohenheim.de (A.C.-S.); martin.hasselmann@uni-hohenheim.de (M.H.); Korinna.Huber@uni-hohenheim.de (K.H.); jseifert@uni-hohenheim.de (J.S.); volker.stefanski@uni-hohenheim.de (V.S.); 2 Institute for Genome Biology, Leibniz Institute for Farm Animal Biology, 18196 Dummerstorf, Germany; omotoso@fbn-dummerstorf.de (A.O.O.); oster@fbn-dummerstorf.de (M.O.); reyer@fbn-dummerstorf.de (H.R.); ponsuksili@fbn-dummerstorf.de (S.P.); wimmers@fbn-dummerstorf.de (K.W.)

**Keywords:** calcium, laying hen, *myo*-inositol, phosphorus, phytate degradation, transcellular mineral transport, transcriptional responses

## Abstract

**Simple Summary:**

Phosphorus is stored as phytate in plant seeds, which are the main components of poultry feed. Poultry can utilize phytate phosphorus after its cleavage catalyzed by enzymes. These enzymes are inhibited by high concentrations of calcium and phosphorus in diets. As laying hens require a high calcium concentration for eggshell production, the inhibition of enzymes might be high. Therefore, the objective of this study was to characterize the degradation of phytate and the utilization of phosphorus and calcium by two laying hen strains supplied with standard or reduced levels of dietary calcium and phosphorus at the egg production peak. The calcium level had a clear effect on phytate degradation products and mineral utilization. The phosphorus level had no effect on these traits, suggesting that actual recommendations for phosphorus supply of laying hens are too high. Differences were noted between the two hen strains and among individual hens regarding calcium and phosphorus metabolism. This is a first step in identifying individual birds that are more capable than others in using phytate phosphorus under challenging conditions. In the long term, our results could help to further reduce the mineral content of laying hen diets.

**Abstract:**

Laying hens require less phosphorus (P) but markedly more calcium (Ca) in their diet than broilers. These differences may cause more distinct interactions with phytate degradation and utilization of minerals in laying hens than those in broilers. The objective of the study was to characterize intestinal phytate degradation, ileal transcript copy numbers of transcellular Ca and P transporters, and mineral utilization by two laying hen strains fed with standard or reduced levels of dietary Ca and P at the laying peak. The strains showed differences regarding several traits driving Ca and P metabolism along the digestive tract. Thus, the two strains may use different mechanisms to meet their respective P demand, i.e., via effective phytate degradation and transcellular transport. Clear effects of the Ca level on *myo*-inositol concentrations and mineral utilization revealed the significance of this element for the measured traits. The absence of P-mediated effects confirmed the findings of several studies recommending that P concentrations used in laying hen feeds are too high. Differences were noted between individuals within one treatment. The next step would be to evaluate the data in individual birds to identify birds that better cope with a challenging diet.

## 1. Introduction

Phosphorus (P) is an essential element for animals, which must be continuously supplied with the feed. Usually, mineral feed phosphates are derived from phosphate rock, a non-renewable resource. Therefore, the optimization of the P supply of farm animals is highly relevant for more sustainable production systems and implies a better understanding of the factors influencing plant-P utilization. In plant feedstuffs, most of the P is present in the form of phytic acid (*myo*-inositol 1,2,3,4,5,6-hexakis (dihydrogen phosphate); InsP_6_) and its salts (phytate). Phytases and other phosphatases are needed to catalyze the cleavage of P from the inositol ring before the released P can be absorbed in the animal intestine. The stepwise cleavage of P yields isomers of lower inositol phosphates with different degrees of phosphorylation (InsP_x_) as intermediate products and *myo*-inositol (MI) as the end product. Contrary to the assumption that non-ruminants are unable to use InsP_6_-P owing to a lack of enzymes, recent studies have shown substantial prececal InsP_6_ degradation (62% to 74%) in the digestive tract of broiler chickens when diets without a phytase supplement and with low P and Ca concentrations were used [1,2,3,4]. The endogenous InsP_6_ degradation in broiler chickens was reduced after feed supplementation of mineral P and additional Ca [1,2,4,5].

The intestinal absorption capacity of both P and Ca in laying hens relies on both transcellular (active) and paracellular (passive) transport mechanisms [6,7]. The transcellular transport of P and Ca is interlinked with the transcellular sodium flux and is influenced by the dietary mineral supply. Moreover, the expression of genes mediating transcellular P and Ca transport in the small intestine of broiler chickens was found to be increased with reduced dietary P and Ca levels [8].

Laying hens require less P in their diet than broiler chickens [9] but need markedly more Ca owing to eggshell formation. These differences may cause more distinct interactions with InsP_6_ degradation, MI release, and P and Ca utilization than in broiler chickens. The reduction of mineral P supplementation in the diet increased ‘phytate digestibility’ by hens from 23% to 48% in the study by Hughes et al. [10], and the effect was not different between two strains of White Leghorn hens. However, significant differences were found by Abudabos [11] between two Hy-Line hen strains with regard to the total tract InsP_6_ degradation (33% vs. 38%). Traits related to mineral utilization have been previously studied in Lohmann Brown-Classic (LB) and Lohmann LSL-Classic (LSL) laying hens using bone strength as a proxy trait [12], whereby LB hens were found to have a higher humerus breaking strength than LSL hens. Strain effects on traits related to InsP degradation were found in a recent study [13]. These effects were partly dependent on the production stage of the hens and therefore probably also on the dietary Ca level.

The objective of this study was to characterize intestinal phytate degradation and mineral utilization by two laying hen strains supplied with standard or reduced levels of dietary Ca and P at the laying peak. The InsP_6_ degradation products and ileal transcript copy numbers of transcellular Ca and P transporters, as well as the features of mineral utilization were measured in individual birds. The hypotheses of this study were as follows:

1. Different P and Ca concentrations in the feed affect InsP_6_ degradation, InsP_x_ pattern, MI concentration, and mineral transporter expression in the digestive tract and MI concentration in the blood and eggs of laying hens.
2. The genetic background of the hens influences the assessed traits.

## 2. Materials and Methods

The study was performed at the Agricultural Experiment Station of the University of Hohenheim, Germany. It was approved by the Regierungspräsidium Tübingen, Germany (Project no., HOH50/17TE) in accordance with the German Animal Welfare Legislation.

### 2.1. Birds and Housing

Hatchlings of brown (LB) and white (LSL) layer strains, representing two distinct genetic backgrounds, were obtained from a breeding company (Lohmann Tierzucht GmbH, Cuxhaven, Germany). For each strain, 16 non-related roosters had been used for egg production. In total, 158 LB and 144 LSL hatchlings were raised in floor pens on deep litter bedding before being moved to metabolic units at the age of 27 weeks. During rearing, the standard management conditions for the pullet phase of the experiment station were applied. The hens received corn-soybean meal-based diets meeting or exceeding the supply recommendations of the breeding company for the starter, grower, pre-laying, and layer phases. During rearing, all hens were weighed at intervals to monitor body weight (BW) development. Prior to the experimental phase, the offspring of 10 roosters per strain were chosen based on the average BW of the hens. At week 27, four randomly chosen offspring of each of the 10 roosters per strain were placed individually in metabolism units (1 × 1 × 1 m), which were distributed in the barn in three connected rooms without doors, in a completely randomized block design, resulting in 10 replicates per strain and dietary treatment. Metabolism units were equipped with a wooden perch, a nest, a feeding trough, water cups, and a wired mesh floor. Stainless steel trays were installed under the units to allow for total excreta collection. A photoperiod of 16-h of light and 8-h of darkness was provided during the experimental period, and the temperature in the barn was set to 18 °C.

### 2.2. Diets

The experimental diets were based on corn and soybean meal to minimize plant intrinsic phytase activity (Table 1). Diets were calculated to contain adequate levels of all nutrients according to the recommendations of the Gesellschaft für Ernährungsphysiologie (GfE) [14], with the exception of Ca and P. Diets contained TiO_2_ as an indigestible marker and were provided in mash form. Diets were mixed in the certified feed mill of the Agricultural Experiment Station of the University of Hohenheim. The experiment was designed as a 2 × 2 × 2 factorial arrangement of treatments (2 laying hen strains, 2 Ca levels, and 2 P levels) and included diets with a standard (39.6 g/kg dry matter [DM]; Ca+) or reduced (33.9 g/kg DM; Ca-) Ca concentration and a standard (5.3 g/kg DM; P+) or reduced (4.7 g/kg DM; P-) P concentration. Feed and water were provided for ad libitum consumption. The calculated nutrient concentrations were confirmed by analyses (Table 1).

### 2.3. Experimental Procedures, Sampling, and Measurements

The experimental diets were provided to the hens after placing them in the metabolism units at week 27. Samples of each diet were obtained before and after the excreta collection phase to determine the DM content of the diets. The animals were monitored at least twice daily. Feed was weighed at the beginning and feed and birds were weighed at the end of the excreta collection phase. In week 30, total excreta were collected from the trays at 24-h intervals for four consecutive days. Feathers, skin scales, and spilled feed were carefully removed before each excreta collection. Feed residues from the trays were weighed, and feed intake was corrected for these residues. Excreta were immediately frozen after each collection at −20 °C. During the excreta collection phase, the eggs of all hens were collected and maintained at room temperature until further processing.

In week 31, the blood and intestinal contents were sampled on four consecutive days beginning at 9 AM each day, whereby treatments were equally distributed among the days. Feed was deprived for 2 h before slaughtering followed by 1 h ad libitum access to the feed to standardize intestinal fill. The hens were individually stunned with a gas mixture of 35% CO_2_, 35% N_2_, and 30% O_2_ and killed by decapitation. The trunk blood was collected in tubes containing sodium fluoride for MI analysis or lithium heparin for P and Ca analysis. Blood samples were then centrifuged for 10 min at 2500 × *g* to obtain the plasma. Digesta from the crop, gizzard, jejunum, the terminal part of the ileum, defined as the last two thirds of the section between Meckel’s diverticulum and 2 cm prior to the ileo-ceco-colonic junction, and both ceca were collected. The crop and gizzard were clamped with an arterial clamp to prevent emptying and were then opened and upended. The crop and gizzard digesta was gently removed with a spatula without scraping the mucosa. The jejunum, terminal ileum, and ceca were cut lengthwise, and digesta was gently removed with a spatula without scraping the mucosa. Digesta samples were immediately frozen at −20 °C, freeze-dried, and pulverized (PULVERISETTE 9; Fritsch GmbH, Idar-Oberstein, Germany). Pulverized samples were stored in airtight containers until further analysis.

From each bird a 2 cm long segment of the ileum was sampled 10 cm distal to the Meckel’s diverticulum. This section was cut open and rinsed with NaCl (0.9%). The ileal mucosa scrapings were retrieved, frozen on dry ice, and stored at −80 °C until RNA extraction. Other samples from different tissues were obtained for other measurements which will be published separately.

Yolk and albumen from one egg per hen were separated, weighed, freeze-dried, weighed again, pulverized with pestle and mortar, and stored until further analysis.

### 2.4. Sample Preparation and Chemical and Physical Analysis

Excreta samples were thawed at 3 °C, weighed, pooled for each individual hen, and homogenized. The excreta DM was analyzed in triplicate. A subsample of the excreta was freeze-dried, and pulverized (PULVERISETTE 9, Fritsch GmbH, Idar-Oberstein, Germany). Pulverized samples were stored in airtight containers until further analysis. Feed ground to pass through a 0.5 mm sieve (Ultra Centrifugal Mill ZM 200; Retsch GmbH, Haan, Germany) and excreta samples were analyzed for DM according to the official method (no. 3.1) [15].

Pulverized feed, digesta, and excreta samples were analyzed for P, Ca, and Ti concentrations by inductively coupled plasma-optical emission spectrometry following wet digestion, using the modified method of Boguhn et al. [16] described in detail by Zeller et al. [3]. Results are provided as g/kg freeze-dried material. Calcium and P utilization was calculated as the proportion of intake that was not recovered in excreta.

The extraction and measurement of InsP_3-6_ isomers in feed and digesta were carried out using the method of Zeller et al. [3] with modifications described by Sommerfeld et al. [5] and high-performance ion chromatography (ICS-3000 system Dionex, Idstein, Germany). By using this method, the separation of enantiomers is not possible; therefore, we were unable to distinguish between the D- and L-forms. Some InsP_3_ isomers could not be identified because the standards were unavailable. A clear discrimination of the isomers Ins(1,2,6)P_3_, Ins(1,4,5)P_3_, and Ins(2,4,5)P_3_ was not possible because of co-elution. Therefore, in this study, we used the term InsP_3x_ for the InsP_3_ isomers of unknown proportion. Results are provided as µmol/g freeze-dried material.

The concentrations of MI in the feed, digesta, yolk, albumen, and blood plasma were analyzed according to Sommerfeld et al. [5] using a gas chromatograph-mass spectrometer after derivatization of the samples. Results in digesta are given as µmol/g freeze-dried material. The results in the albumen and yolk are given as µmol/g original material. The total MI content in the albumen or yolk was calculated based on the MI concentration in the freeze-dried material and its mass. The MI content in the egg (without shell) was calculated as the sum of the MI contents in the egg yolk and albumen.

Calcium and inorganic P (P_i_) in blood plasma were analyzed at the IDEXX BioResearch Vet Med Labor GmbH (Ludwigsburg, Germany). Calcium was measured photometrically by the Arsenazo method using Beckman Olympus AU480. Inorganic P was measured photometrically as a phosphomolybdate complex in a Beckman Olympus AU480.

The particle sizes of the limestone samples were measured by wet sieving analysis using a sieve shaker (AS200, Retsch GmbH, Germany) with sieve sizes of 2.000, 1.180, 1.000, 0.500, 0.250, 0.125, and 0.063 mm, as described by Grubješić et al. [17].

### 2.5. Total RNA Isolation and cDNA Synthesis

Total RNA was extracted from the tissue samples using TRizol reagent (Sigma-Aldrich, Taufkirchen, Germany) with strict adherence to the manufacturer’s guidelines. The follow-up processing comprised a DNase I treatment and purification using the column-based RNeasy Mini Spin Kit (Qiagen GmbH, Hilden, Germany). The assessment of RNA quality and the measurement of concentration was performed using a NanoDrop ND-2000 spectrophotometer (Thermo Fisher Scientific, Dreieich, Germany). Subsequently, cDNA was synthesized using 1500 ng of RNA, along with random primers (Promega, Mannheim, Germany), oligo d(T) nucleotides, and RNasin plus (Promega), in the presence of SuperScript III Reverse Transcriptase (Invitrogen, Karlsruhe, Germany) according to the manufacturer’s instructions. The cDNA samples were diluted with distilled water to a final volume of 200 µL and stored at −20 °C. The absence of genomic DNA contamination in the converted cDNA was verified by polymerase chain reaction (PCR) with an intron spanning primer set for chicken GAPDH employing SupraTherm Taq DNA polymerase (GeneCraft, Münster, Germany).

### 2.6. Quantitative Real-Time PCR (qRT-PCR)

Gene expression profiling was performed for selected genes encoding for Ca and P transporters: ATPase plasma membrane Ca2+ transporting 1 (ATP2B1), Ca binding protein 1 (CALB1), sodium-Ca exchanger member 1 (NCX1), solute carrier family 20 member 1 (SLC20A1), solute carrier family 20 member 2 (SLC20A2), and solute carrier family 34 member 2 (SLC34A2). GAPDH and ACTB were used as housekeeping genes. The gene-specific primers, corresponding annealing temperatures, and resulting fragment lengths are described in Supplementary Table S1. The cDNA samples (*n* = 10 per dietary group and strain) were analyzed in duplicate via the Light Cycler 480 system using the Light Cycler 480 SYBR Green I Master mix (Roche, Mannheim, Germany) according to the manufacturer’s guidelines. Reactions were performed in a final volume of 12 µL using the LightCycler 480 SYBR Green I Master mix (Roche) and gene-specific primers (Supplementary Table S1). The temperature profiles included an initial denaturation step at 95 °C for 5 min, followed by 45 cycles comprising denaturation at 95 °C for 10 s, annealing at 60 °C for 15 s, and extension/fluorescence acquisition at 72 °C for 25 s. The specificity of amplification products was assessed by melting curve analysis. For all the assays, threshold cycles were converted to copy numbers of respective transcripts using a standard curve generated by amplifying serial dilutions of the corresponding PCR standard (108–101 copies).

### 2.7. Statistical Analysis

All data were subjected to a 3-factorial analysis of variance using the MIXED procedure and pairwise *t*-tests using the software package SAS (version 9.3; SAS Institute Inc., Cary, North Carolina). Data without normal distribution were either log or square root transformed. The results are presented as the LSmeans and pooled SEM of the untransformed data. The individual hen was considered as the experimental unit. The following model was used:

Y_ijklmn_ = µ + α_i_ + β_j_ + γ_k_ + (αβ)_ij_ + (αγ)_ik_ + (βγ)_jk_ + (αβγ)_ijk_ + δ_l_ + φ_m_ + χ_n_ + ε_ijklmn_, (1)

where Y_ijklmn_ = response variable; µ = overall mean; α_i_ = effect of strain (fixed); β_j_ = effect of dietary P (fixed); γ_k_ = effect of dietary Ca (fixed), all possible interactions among strains, dietary P, and dietary Ca (fixed); δ_l_ = room (fixed); φ_m_ = block (random), which includes the different sampling days; χ_n_ = father/rooster (random), and ε_ijklmn_ = residual error. Statistical significance was declared at *p* < 0.05.

## 3. Results

### 3.1. Performance and Blood Traits

The BW of hens at week 30 was significantly affected by the interaction of strain × P × Ca (*p* = 0.016; Table 2). LB hens fed P+Ca− feeds were significantly heavier than LB hens fed P+Ca+ or P−Ca− feeds. The BW of LSL hens did not differ among dietary treatments. Irrespective of the strain, the average daily feed intake was significantly lower when hens were fed Ca+ feed (*p* = 0.008). In the excreta collection period, the average egg weight of LSL hens was higher than that of LB hens (*p* = 0.002) and was higher with P− than P+ treatments (*p* = 0.047). The concentrations of P_i_, Ca, and MI in blood plasma did not significantly differ among treatments (*p* > 0.05).

### 3.2. InsP6, Inositol Phosphate Isomers, and MI in Gut Sections

#### 3.2.1. Crop

InsP_6_ and MI concentrations in the crop did not differ among treatments (*p* > 0.05; Table 3). The concentration of Ins(1,2,3,4,5)P_5_ was significantly lower in LB hens fed P+ feed (0.3 µmol/g) than in LB hens fed other diets (0.4 µmol/g), resulting in a strain × P interaction (*p* = 0.025). The concentration of Ins(1,2,4,5,6)P_5_ was higher in LSL hens (0.9 µmol/g) than in LB hens (0.8 µmol/g) (*p* = 0.019).

#### 3.2.2. Gizzard

The concentrations of InsP_6_ and Ins(1,2,4,5,6)P_5_ in the gizzard were significantly higher in LB hens than in LSL hens (*p* ≤ 0.005; Table 3). The MI concentration was significantly lower in LSL hens fed Ca+ feed (0.34 µmol/g) than in all other treatments, resulting in a strain × Ca interaction (*p* = 0.010).

#### 3.2.3. Jejunum

The concentrations of InsP_6_, Ins(1,2,3,4,5)P_5_, and Ins(1,2,4,5,6)P_5_ in the jejunum were significantly higher in LB hens than in LSL hens (*p* ≤ 0.049; Table 4). The Ins(1,2,4,5,6)P_5_ concentration was higher in the Ca+ treatments (*p* = 0.037). The MI concentration was affected by Ca as a trend (*p* = 0.054), with lower concentrations in Ca+ treatments.

#### 3.2.4. Ileum

Most InsP isomers in the ileum were not affected by strain or diet (Table 5). However, the concentration of Ins(1,2,3,4)P_4_ was significantly higher in LSL hens than in LB hens. The MI concentration was significantly lower in Ca+ than in Ca− treatments (*p* = 0.001).

#### 3.2.5. Ceca

The concentrations of InsP_6_, Ins(1,2,3,4,5)P_5_, and Ins(1,2,3,4)P_4_ in the ceca were significantly higher in LB hens than in LSL hens (*p* ≤ 0.024; Table 6). The concentrations of Ins(1,2,3,4,5)P_5_ and Ins(1,2,3,4)P_4_ were lower in Ca− treatments than in Ca+ treatments (*p* ≤ 0.045). The MI concentration in the ceca was significantly affected by a three-way-interaction (*p* = 0.049). It was the highest in LSL hens receiving the P−Ca+ feed and significantly lower in LSL hens receiving the P+Ca+ feed, and LB hens irrespective of the diet.

### 3.3. Ca and P Intake, Concentration in the Small Intestine, and Utilization

The daily Ca intake (*p* = 0.004), Ca concentration in the jejunum (*p* = 0.027) and ileum (*p* = 0.014), and Ca excretion (*p* < 0.001) increased and Ca utilization significantly decreased (*p* < 0.001) with higher Ca concentration in feeds (Table 7). Higher P concentration in feeds increased the daily P intake and P excretion (*p* < 0.001). The daily P intake (*p* < 0.001) and P utilization (*p* = 0.013) were lower in hens receiving the Ca+ than in hens receiving Ca− treatments. The P concentrations in the jejunum and ileum were not significantly affected by the treatments or their interactions.

### 3.4. Expression Pattern of Transcellular Mineral Transporters in the Ileum

In the ileum, genes encoding transcellular P transport, such as SLC20A1 and SLC34A2, exhibited a clear strain effect (Table 8) as the transcript abundances were significantly higher in LB hens than in LSL hens (FC = 1.60 and 1.34, respectively; *p* ≤ 0.007). SLC20A2 expression was higher in LB hens receiving P+ feed than in LSL hens receiving P+ feed (FC = 1.27), resulting in a significant strain × P interaction (*p* = 0.027). SLC34A2 expression tended to be higher in hens receiving Ca− feed than in those receiving Ca+ feed (FC = 1.20). Regarding genes encoding transcellular Ca transport in the ileum, LB hens tended to have 24% higher and 25% lower expressions of ATP2B1 (*p* = 0.077) and NCX1 (*p* = 0.083), respectively, than LSL hens. CALB1 expression tended to be higher in LSL hens receiving P− feeds than in LB hens receiving P− feeds (FC = 1.53). The other treatments did not differ, resulting in a strain × P interaction (*p* = 0.067). CALB1 expression tended to be 19% higher in hens receiving Ca− feed than in hens receiving Ca+ feed (*p* = 0.090).

### 3.5. MI in the Egg

The highest MI concentration in the egg yolk was found in LB hens receiving P−Ca+ feed (Table 9). LB hens receiving P+Ca+ feed and LSL hens receiving P−Ca+ feed had significantly lower MI concentrations, and all other treatments resulted in in-between concentrations, causing a three-way interaction (*p* = 0.023). The MI content in the egg yolk was significantly lower in LB hens receiving P+Ca+ feed than in hens receiving other treatments, except for LB hens receiving P−Ca− feed, resulting in an interaction (*p* = 0.025). The MI concentration or content in the albumen or MI content in the whole egg did not differ among treatments.

## 4. Discussion

One hypothesis of this experiment was that the dietary P and Ca levels influence the InsP degradation pattern in the digestive tract and P and Ca utilization by laying hens. As P utilization has been shown to be a heritable trait in Japanese quail and broiler chickens [18,19,20], we further hypothesized that different hen strains and thus genetic backgrounds have an influence.

### 4.1. Strain Effects

**Figure 1:**
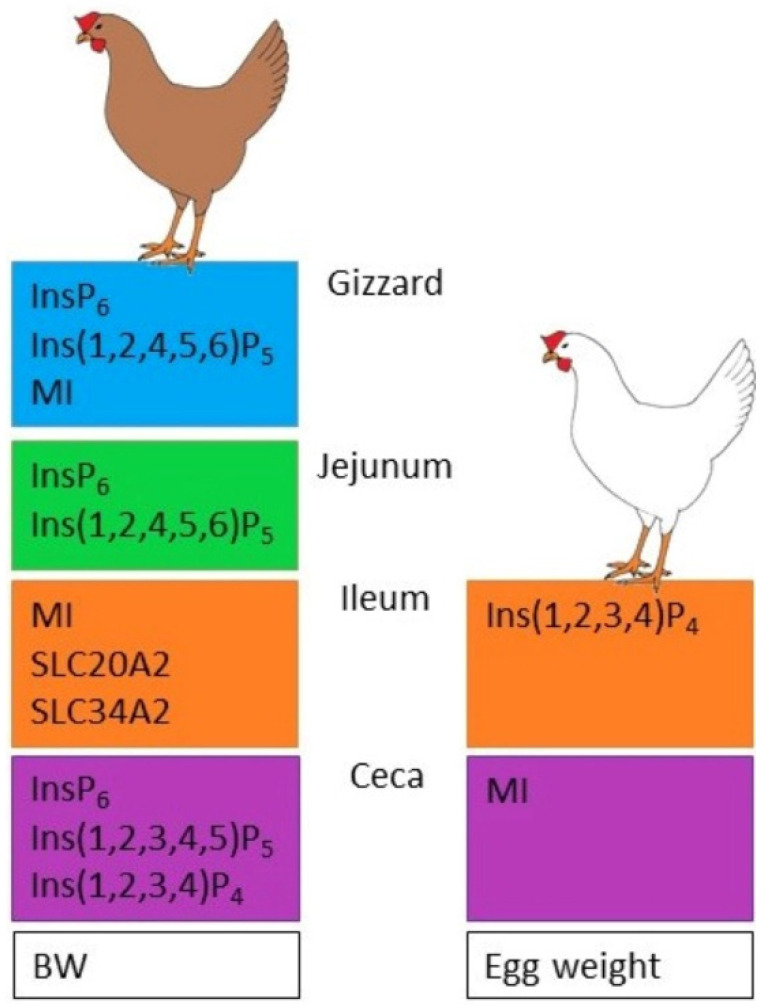
Summary of traits that were shown to be significantly higher in one laying hen strain than in the other.

Strain effects were observed for several traits driving Ca and P metabolism along the digestive tract (Figure 1). In the gizzard, LB hens had higher concentrations of InsP_6_ and Ins(1,2,4,5,6)P_5_ than did LSL hens. This might indicate a slower degradation of InsP_6_ in the anterior digestive tract by LB hens or a different passage rate of small particles through the gizzard between the strains. The significantly lower MI concentration in LSL hens fed Ca+ diets, which was not observed in LB hens, indicated that the LSL strain reacted more sensitively to different dietary Ca levels. A very high Ca level (23.3 g/kg) compared to a standard level (10.7 g Ca/kg) significantly increased the pH of crop and ileum content of broiler chickens [21]. Thus, the contrasting reaction to the higher Ca level between the hen strains might be due to the pH changes affecting the strains differently or indicating a strain-specific Ca requirement in the diet.

Strain effects were also observed in the jejunum, with LB hens having higher concentrations of InsP_6_, Ins(1,2,4,5,6)P_5_, and Ins(1,2,3,4,5)P_5_ than LSL hens. This confirms the assumption of differences in InsP_6_ degradation between the two strains, as has already been shown by Abudabos [11] with two other hen strains. Additionally, LB hens revealed significantly higher expression rates of two sodium/phosphate co-transporters (SLC20A1, SLC34A2) in the ileum than LSL hens. SLC34A2 plays an important role in the transcellular P transport mechanism along the intestine in rodents and human [22,23] while its RNA expression was low in pigs [24]. In laying hens, SLC34A2 showed a remarkable mRNA copy number in the ileum, suggesting that it is a relevant transcellular P transporter in the intestine of poultry. Correspondingly, SLC20A1 has been reported to be expressed in the mid and posterior intestine, including the ileum, underlining its contribution to P absorption in this segment [25]. In view of the strain-specific intestinal capacity of phytate degradation in LB and LSL laying hens, the results of the ileal mineral P transporters suggest compensatory mechanisms in LB hens to balance P absorption.

**Figure 2:**
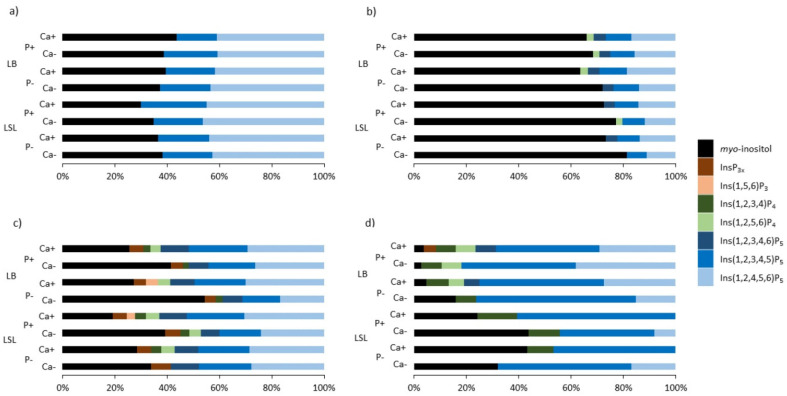
Relative proportions of the concentrations of InsP_3-5_ isomers and *myo*-inositol in the digesta of the crop (**a**), jejunum (**b**), terminal ileum (**c**), and ceca (**d**) of two laying hen strains (LB and LSL) aged 31 weeks. The sum of the concentrations of InsP_3-5_ isomers and *myo*-inositol on a molar basis is defined as 100%. The InsP pattern in the crop is mainly derived from the InsP present in the feedstuff. The pattern became more diverse in the jejunum and even more in the ileum before it got less diverse in the ceca.

Significantly higher concentrations of InsP_6_, Ins(1,2,3,4,5)P_5_, and Ins(1,2,3,4)P_4_ and lower concentrations of MI in the ceca of LB hens compared to that in LSL hens seem to confirm the assumption of Sommerfeld et al. [13] that these strains might differ in their intestinal morphology, leading to different degrees of InsP degradation. What both strains had in common is that the InsP pattern became more diverse from the jejunum to the ileum (Figure 2), and the relative proportion of MI and other InsP isomers in the sum of all InsPs changed along the digestive tract.

In the crop, one of the InsP_5_ isomers was affected by strain and the other by strain × P. However, the detected isomers likely originated from the feed (Table 1) and the numerical differences were negligibly small. The concentrations of InsP_6_ and MI in the crop were not affected by the treatments, which is another indication for negligible phytase activity in the crop of laying hens, which has already been proposed by Marounek et al. [26]. Phytase activity in the crop contents of the 38- or 47-week old laying hens was lower than that in the small intestine when measured as activity per segment [26,27]. However, phytase activity in the crop content was identical to that of the small intestine of laying hens aged 47 weeks when measured per gram of digesta [26,27]. Possibly, the relatively short retention time in the crop when feed is offered for ad libitum consumption [28,29] leads to an insignificant degradation of InsP_6_.

In this study, we also analyzed the concentrations of the indigestible marker TiO_2_ along the digestive tract and its total excretion. The TiO_2_ concentrations in the gizzard, jejunum, terminal ileum, and ceca were significantly higher in LB hens than in LSL hens, but the TiO_2_ recovery in the excreta was not (Supplementary Table S2). This indicates that the passage rate of different constituents of the feed differed between the two strains. The recovery ranged from 88% to 95% in all treatments. In the study by Peddie et al. [30], the daily recovery of TiO_2_ ranged between 76% and 145% in the excreta of individual laying hens over several days. It was speculated that these variations derived from methodological inaccuracies in sampling feed and excreta for individual birds over a short time. In the present study, the feed being in mash form possibly made it harder to collect than pellets when spilled and, coupled with the relatively low amount of excreta, led to some inaccuracies and the recovery deviating from 100%.

Not only differences between the strains were observed, but also remarkable differences between individuals within different treatments. Similar observations, given by the high standard deviations for all measured traits among individual hens, were found by Marounek et al. [26,27].

### 4.2. Effects of Dietary Ca and P

The MI concentrations in the jejunum as well as in the ileum were lower when Ca+ diets were fed, and more Ca was present in the digesta. Possibly, Ca had a diminishing effect on endogenous phosphatases promoting the degradation of lower InsP to MI. Consistent with this assumption, a decreased activity of alkaline phosphatase and phytase in duodenal mucosa of broiler chickens with increased dietary Ca was found [31,32]. In the present study, the MI concentration in the jejunum and the MI content in egg yolk were positively related (r = 0.872; *p* = 0.005). However, no relationship between MI content in the egg yolk and MI concentration in the ileum was found. This is in line with the results of Sommerfeld et al. [13], who found a similar relationship, which might indicate the jejunum being the main absorption site of MI in the digestive tract [33].

With more Ca in the diet, hens ingested and excreted more Ca. A greater difference between Ca− and Ca+ in excretion than intake resulted in a lower percentage Ca utilization with Ca+. A higher Ca utilization at a lower dietary Ca concentration has already been shown by Rodehutscord et al. [34]. It seems that the absorption and use efficiency of dietary Ca increases when less Ca is provided [35,36]. Accordingly, the ileal Ca transporter CALB1 tended to be higher expressed with lower dietary Ca levels. Studies on chicken with reduced dietary Ca concentration showed a significant increase in duodenal CALB1 mRNA levels [8] suggesting that CALB1 is part of the compensatory response to variable dietary Ca levels.

A higher dietary P level led to a higher P intake and a higher P excretion. The utilization of P, however, was not influenced by the dietary P level. Nonetheless, due to the complex regulatory mechanisms in mineral metabolism, including in the intestine, bone, and kidney [37], the elevated dietary Ca contents decreased both P intake and P utilization, as additionally reflected in the numerically lower mRNA copy numbers of SLC34A2 between laying hens fed high and low dietary Ca contents. The lower P intake could be explained by the general lower feed intake due to the higher dietary Ca concentration. The P excretion was not affected by Ca, which means that, although less P reached the small intestine, the same amount was excreted. This could indicate that the excess Ca in the Ca+ treatments formed complexes with P that were then not absorbable and thus excreted, leading to a lower P utilization. Supporting evidence for a Ca−P−complex formation in the small intestine is provided by the P concentrations in the jejunum and ileum, which were not, as expected, lower in the P− treatments, but instead did not differ among treatments. The observation that P utilization was only influenced by dietary Ca level and not by P level is consistent with a previous study [34] and might be an indication for Ca not only altering the P intake level, but also the absorption process of P.

Although effects of the diets on the utilization of Ca and P were observed in this study, there was no effect on the plasma Ca and P concentrations. No effects of increasing P levels (from 3.8 to 6.1 g P/kg feed) on plasma mineral concentrations were found by Boorman and Gunaratne [38] feeding wheat-soybean meal-based diets to 37-week-old ISA brown hens. However, the plasma P was significantly increased with excess dietary P (13.9 g P/kg feed) and dependent of the time of oviposition and whether an egg was laid or not on the day of blood sampling. Jing et al. [39] measured lower plasma P concentrations when Lohmann LSL-classic hens were fed corn-soybean meal-based diets containing 0.38% P compared to 0.63% P or higher. Plasma Ca concentrations did not differ among these treatments. As a proportion of the total Ca measured in the blood is bound and not available for eggshell formation [40], it might be recommended to analyze ionized Ca in future experiments. One supportive previous finding is that of Frost and Roland [40], who observed a dietary P effect on ionized Ca in the plasma, but not on total Ca.

## 5. Conclusions

The effects of Ca concentration on MI concentrations and P and Ca utilization revealed the significance of this element for the measured traits. Moreover, the absence of P-mediated effects could be attributed to the fact that the current feeding regimes do not necessarily reflect the effective P requirement of both LB and LSL laying hens. Thus, LB and LSL strains possibly employ different mechanisms to meet their respective P demand, i.e., via effective phytate degradation (LSL) and transcellular transport (LB). Therefore, a corresponding P reduction of 20% is feasible in laying hens. This confirms the findings of several studies that suggest that the recommendations for P supply of laying hens are too high [9,39,41].

As a consequence of the remarkable differences between individuals within treatments in this study, the next step would be to evaluate our data together with data by companion projects on an individual hen basis. From this, it might be possible to find individuals coping better with a challenging diet than others which could help to further reduce the mineral content of laying hen diets. In the long term, this will contribute to feeding strategies without using mineral phosphates and thereby maintaining the finite global rock phosphate stores.

## Figures and Tables

**Table 1:** Ingredient composition and calculated and analyzed nutrient concentrations of the experimental diets.

Ingredient, g/kg	P+Ca+	P+Ca−	P−Ca+	P−Ca−
Corn	617.4	617.4	617.4	617.4
Soybean meal	265.0	265.0	265.0	265.0
Soybean oil	12.0	12.0	12.0	12.0
DL-Methionine	3.5	3.5	3.5	3.5
Monocalcium phosphate	5.3	5.3	3.0	3.0
Sand	-	12.8	1.3	14.1
Limestone, fine ^1^	24.7	21.3	25.2	21.7
Limestone, coarse ^2^	58.7	49.3	59.2	50.0
Sodium chloride	3.0	3.0	3.0	3.0
Choline chloride	1.0	1.0	1.0	1.0
Sodium bicarbonate	1.9	1.9	1.9	1.9
Vitamin mix ^3^	2.0	2.0	2.0	2.0
Mineral mix ^4^	0.5	0.5	0.5	0.5
TiO_2_	5.0	5.0	5.0	5.0
**Calculated concentration**				
Crude protein, g/kg DM	189	189	189	189
Total P, g/kg DM	5.3	5.3	4.7	4.7
Non-phytate P, g/kg DM	2.9	2.9	2.3	2.3
Ca, g/kg DM	39.6	33.9	39.6	33.9
**Analyzed concentration** ^5^				
Total P, g/kg DM	5.3	5.3	4.7	4.7
InsP_6_-P, g/kg DM	2.3	2.2	2.4	2.4
Ca, g/kg DM	39.5	35.1	40.3	34.4
*Myo*-inositol, µmol/g DM	1.1	1.1	1.1	1.1
Ins(1,2,3,4,5)P_5_, µmol/g DM	0.5	0.5	0.5	0.5
Ins(1,2,4,5,6)P_5_, µmol/g DM	1.0	1.0	1.1	1.0
InsP_6_, µmol/g DM	12.6	12.0	12.8	12.7

^1^ Particle size: >0.25 mm, 0.9%; 0.125–0.25 mm, 3.3%; 0.063–0.125 mm, 11.0%; <0.063 mm, 84.8%. ^2^ Particle size: >1.18 mm, 12.3%; 1–1.18 mm, 34.8%; 0.5–1 mm, 48.8%; 0.063–0.5 mm, 0.6%; <0.063 mm, 3.6%. ^3^ Vitamin premix (Miavit GmbH, Essen, Germany), provided per kg of complete diet: 10,000 IU vitamin A, 3000 IU vitamin D3, 30 mg vitamin E, 2.4 mg vitamin K3, 100 µg biotin, 1 mg folic acid, 3 mg vitamin B1, 6 mg vitamin B2, 6 mg vitamin B6, 30 µg vitamin B12, 50 mg nicotinamide, and 14 mg calcium-D-pantothenat. ^4^ Trace element premix (Gelamin Gesellschaft für Tierernährung mbH, Memmingen, Germany), provided per kg of complete diet: 80 mg manganese from manganese-(II)-oxide, 60 mg zinc from zinc-oxide, 25 mg iron from ferrous-(II)-sulphate monohydrate, 7.5 mg copper from cupric-(II)-sulphate pentahydrate, 0.6 mg iodine from calcium iodate, 0.2 mg selenium from sodium selenite. ^5^ All other InsP isomers were below the limit of quantification or limit of detection.

**Table 2:** Effect of hen strain, P, and Ca on body weight (BW); average daily feed intake (ADFI); average egg weight in the excreta collection period; and concentrations of inorganic P, Ca, and *myo*-inositol in the blood plasma of laying hens aged 31 weeks.

			BW	ADFI	Average Egg Weight	Inorganic P	Calcium	*myo*-Inositol
Strain	Dietary P	Dietary Ca	g	g/d	g	mmol/L	mmol/L	mmol/L
LB ^1^	P+	Ca+	1784 ^bc^	104	57.2	1.8	7.5	0.15
LB	P+	Ca−	1929 ^a^	114	58.2	1.7	7.3	0.14
LB	P−	Ca+	1838 ^ab^	111	58.8	1.8	6.9	0.14
LB	P−	Ca−	1809 ^b^	110	59.6	1.8	7.1	0.16
LSL ^2^	P+	Ca+	1648 ^d^	109	60.6	1.5	6.2	0.14
LSL	P+	Ca−	1641 ^d^	116	59.5	1.8	7.3	0.15
LSL	P−	Ca+	1599 ^d^	109	61.2	1.7	7.3	0.15
LSL	P−	Ca−	1683 ^cd^	118	62.6	1.6	6.9	0.15
Pooled SEM			41.0	3.3	1.15	0.11	0.47	0.010
*p-*values	Strain		<0.001	0.165	0.002	0.133	0.425	0.847
	P		0.500	0.571	0.047	0.514	0.857	0.615
	Ca		0.081	0.008	0.474	0.705	0.551	0.458
	Strain × P		0.579	0.798	0.859	0.562	0.261	0.671
	Strain × Ca		0.717	0.387	0.525	0.172	0.634	0.728
	P × Ca		0.445	0.293	0.460	0.312	0.363	0.418
	Strain × P × Ca		0.016	0.202	0.451	0.055	0.121	0.085

Data are given as LSmeans; *n* = 8–10 hens. ^1^ LB = Lohmann Brown-Classic. ^2^ LSL = Lohmann LSL-Classic. ^a–d^ Different superscript letters within a column indicate significant interaction effects among strains, dietary P, and dietary Ca (*p* < 0.05).

**Table 3:** Effect of strain, P, and Ca on concentrations of *myo*-inositol and InsP isomers in the crop and gizzard of laying hens aged 31 weeks (µmol/g).

			Crop				Gizzard			
Strain	Dietary P	Dietary Ca	*myo*-Inositol	Ins(1,2,3,4,5)P_5_	Ins(1,2,4,5,6)P_5_	InsP_6_	*myo*-Inositol	Ins(1,2,3,4,5)P_5_	Ins(1,2,4,5,6)P_5_	InsP_6_
LB ^1^	P+	Ca+	0.85	0.3	0.8	10.6	0.65	<LOQ ^3^	0.5	8.6
LB	P+	Ca−	0.76	0.4	0.8	10.6	0.60	n.d. ^4^	0.4	7.7
LB	P−	Ca+	0.85	0.4	0.9	12.0	0.60	0.2	0.5	8.4
LB	P−	Ca−	0.77	0.4	0.9	11.6	0.65	<LOQ	0.4	7.0
LSL ^2^	P+	Ca+	0.60	0.5	0.9	11.8	0.31	n.d.	0.3	5.8
LSL	P+	Ca−	0.75	0.4	1.0	11.8	0.55	n.d.	<LOQ	5.2
LSL	P−	Ca+	0.75	0.4	0.9	11.9	0.36	n.d.	0.3	5.7
LSL	P−	Ca−	0.80	0.4	0.9	11.8	0.55	0.2	0.3	6.7
Pooled SEM			0.090	0.03	0.06	0.67	0.059	0.04	0.06	0.85
*p-*values	Strain		0.235	0.024	0.019	0.188	0.001	.	0.005	0.002
	P		0.370	0.475	0.153	0.164	0.770	.	0.921	0.762
	Ca		0.833	0.656	0.786	0.840	0.014	.	0.723	0.493
	Strain × P		0.405	0.025	0.056	0.215	0.760	.	0.832	0.292
	Strain × Ca		0.121	0.832	0.433	0.914	0.010	.	0.178	0.258
	P × Ca		0.687	0.681	0.355	0.799	0.760	.	0.979	0.703
	Strain × P × Ca		0.640	0.128	0.750	0.851	0.368	.	.	0.449

Data are given as LSmeans; *n* = 9–10 hens. InsPs other than those reported were not detected. ^1^ LB = Lohmann Brown-Classic. ^2^ LSL = Lohmann LSL-Classic. ^3^ <LOQ, not quantifiable in the majority of samples. ^4^ n.d., not detectable in the majority of samples.

**Table 4:** Effect of strain, P, and Ca on concentrations of *myo*-inositol and InsP isomers in the jejunum of laying hens aged 31 weeks (µmol/g).

Strain	Dietary P	Dietary Ca	*myo*-Inositol	Ins(1,2,5,6)P_4_	Ins(1,2,3,4,6)P_5_	Ins(1,2,3,4,5)P_5_	Ins(1,2,4,5,6)P_5_	InsP_6_
LB ^1^	P+	Ca+	7.40	0.3	0.5	1.1	1.9	27.9
LB	P+	Ca−	8.26	0.3	0.5	1.1	1.9	31.7
LB	P−	Ca+	8.53	0.4	0.6	1.4	2.5	36.1
LB	P−	Ca−	8.82	<LOQ ^3^	0.5	1.2	1.7	32.8
LSL ^2^	P+	Ca+	8.71	<LOQ	0.5	1.1	1.7	28.4
LSL	P+	Ca−	9.16	0.3	<LOQ	1.0	1.4	27.1
LSL	P−	Ca+	8.55	<LOQ	0.5	1.0	1.6	26.3
LSL	P−	Ca−	9.54	n.d. ^4^	<LOQ	0.9	1.3	27.8
Pooled SEM			0.532	0.06	0.06	0.12	0.24	2.33
*p-*values	Strain		0.110	0.704	0.342	0.049	0.021	0.019
	P		0.149	0.445	0.782	0.459	0.848	0.211
	Ca		0.054	0.908	0.634	0.266	0.037	0.906
	Strain × P		0.264	.	0.245	0.219	0.293	0.097
	Strain × Ca		0.824	.	.	0.853	0.701	0.960
	P × Ca		0.982	.	0.213	0.355	0.239	0.499
	Strain × P × Ca		0.397	.	.	0.355	0.203	0.127

Data are given as LSmeans; *n* = 10 hens. InsPs other than those reported were not detected. ^1^ LB = Lohmann Brown-Classic. ^2^ LSL = Lohmann LSL-Classic. ^3^ <LOQ, not quantifiable in the majority of samples. ^4^ n.d., not detectable in the majority of samples.

**Table 5:** Effect of strain, P, and Ca on concentrations of *myo*-inositol and InsP isomers in the ileum of laying hens aged 31 weeks (µmol/g).

Strain	Dietary P	Dietary Ca	*myo*-inositol	InsP_3x_	Ins(1,5,6)P_3_	Ins(1,2,3,4)P_4_	Ins(1,2,5,6)P_4_	Ins(1,2,3,4,6)P_5_	Ins(1,2,3,4,5)P_5_	Ins(1,2,4,5,6)P_5_	InsP_6_
LB ^1^	P+	Ca+	1.93	0.4	n.d. ^3^	0.2	0.3	0.8	1.7	2.2	40.0
LB	P+	Ca−	3.76	0.4	n.d.	0.2	<LOQ ^4^	0.7	1.6	2.4	43.5
LB	P−	Ca+	2.36	0.4	0.4	<LOQ	0.4	0.8	1.7	2.6	46.3
LB	P−	Ca−	6.41	0.5	<LOQ	0.3	<LOQ	0.9	1.7	2.0	46.5
LSL ^2^	P+	Ca+	1.83	0.5	0.3	0.4	0.5	1.0	2.1	2.9	45.6
LSL	P+	Ca−	3.42	0.5	n.d.	0.3	0.4	0.6	1.4	2.1	35.6
LSL	P−	Ca+	2.20	0.4	<LOQ	0.3	0.4	0.7	1.5	2.2	41.6
LSL	P−	Ca−	2.19	0.5	<LOQ	<LOQ	<LOQ	0.7	1.3	1.8	38.4
Pooled SEM			0.794	0.08	0.07	0.07	0.09	0.12	0.20	0.34	4.11
*p-*values	Strain		0.061	0.298	.	0.030	0.507	0.694	0.471	0.752	0.176
	P		0.766	0.488	.	0.605	0.884	0.730	0.352	0.328	0.471
	Ca		0.001	0.433	.	0.449	0.748	0.170	0.076	0.113	0.405
	Strain × P		0.126	0.429	.	.	0.459	0.119	0.247	0.355	0.350
	Strain × Ca		0.449	0.763	.	0.628	.	0.221	0.144	0.352	0.138
	P × Ca		0.710	0.493	.	.	.	0.134	0.368	0.655	0.752
	Strain × P × Ca		0.140	0.968	.	.	.	0.355	0.362	0.229	0.366

Data are given as LSmeans; *n* = 8–10 hens. InsPs other than those reported were not detected. ^1^ LB = Lohmann Brown-Classic. ^2^ LSL = Lohmann LSL-Classic. ^3^ n.d., not detectable in the majority of samples. ^4^ <LOQ, not quantifiable in the majority of samples.

**Table 6:** Effect of strain, P, and Ca on concentrations of *myo*-inositol and InsP isomers in the ceca of laying hens aged 31 weeks (µmol/g).

Strain	Dietary P	Dietary Ca	*myo*-Inositol	InsP_3x_	Ins(1,2,3,4)P_4_	Ins(1,2,5,6)P_4_	Ins(1,2,3,4,6)P_5_	Ins(1,2,3,4,5)P_5_	Ins(1,2,4,5,6)P_5_	InsP_6_
LB ^1^	P+	Ca+	0.24 ^c^	0.3	0.5	0.5	0.5	2.6	1.9	26.0
LB	P+	Ca−	0.15 ^c^	n.d.	0.4	0.4	n.d.	2.3	2.0	25.4
LB	P−	Ca+	0.32 ^c^	n.d.	0.6	0.4	0.4	3.3	1.9	25.6
LB	P−	Ca−	0.42 ^c^	n.d.	0.2	n.d.	n.d.	1.6	0.4	9.5
LSL ^2^	P+	Ca+	0.64 ^bc^	n.d.	0.4	n.d.	n.d.	1.6	n.d.	2.4
LSL	P+	Ca−	1.09 ^ab^	n.d.	0.3	n.d.	n.d.	0.9	0.2	6.1
LSL	P−	Ca+	1.30 ^a^	n.d.	0.3	n.d.	n.d.	1.4	n.d.	3.0
LSL	P−	Ca−	0.94 ^ab^	n.d.	n.d.	n.d.	n.d.	1.5	0.5	5.1
Pooled SEM			0.189	0.08	0.09	0.09	0.12	0.48	0.50	5.79
*p*-values	Strain		<0.001	.	0.024	.	.	0.019	0.119	0.001
	P		0.088	.	0.191	0.454	0.710	0.797	0.598	0.299
	Ca		0.831	.	0.012	0.350	.	0.045	0.115	0.510
	Strain × P		0.752	.	0.472	.	.	0.777	0.099	0.322
	Strain × Ca		0.885	.	0.683	.	.	0.295	.	0.163
	P × Ca		0.214	.	0.089	.	.	0.609	0.071	0.285
	Strain × P × Ca		0.049	.	.		.	0.101	.	0.395

Data are given as LSmeans; *n* = 5–10 hens. InsPs other than those reported were not detected. ^1^ LB = Lohmann Brown-Classic. ^2^ LSL = Lohmann LSL-Classic. ^a–c^ Different superscript letters within a column indicate significant interaction effects among strains, dietary P, and dietary Ca (*p <* 0.05).

**Table 7:** Effect of strain, P, and Ca on Ca and P intake, concentration of Ca and P in the jejunum and ileum, and Ca and P utilization of laying hens at 31 weeks of age.

			Ca Intake	Ca Jejunum	Ca Ileum	Ca Excretion	Ca Utilization	P Intake	P Jejunum	P Ileum	P Excretion	P Utilization
Strain	Dietary P	Dietary Ca	g/d	g/kg	g/kg	g/d	%	g/d	g/kg	g/kg	g/d	%
LB ^1^	P+	Ca+	3.91	27.9	53.2	1.33	66.1	0.53	8.7	11.0	0.39	24.9
LB	P+	Ca−	3.86	22.4	32.2	1.08	72.7	0.58	9.2	11.6	0.41	31.0
LB	P−	Ca+	4.14	27.3	37.2	1.56	63.3	0.49	10.6	12.5	0.36	26.0
LB	P−	Ca−	3.60	21.3	34.1	0.99	72.6	0.50	9.5	12.0	0.37	25.1
LSL ^2^	P+	Ca+	4.09	28.3	44.1	1.49	63.3	0.55	9.1	12.4	0.43	21.9
LSL	P+	Ca−	3.96	27.0	35.0	1.10	72.7	0.60	9.0	11.2	0.43	28.3
LSL	P−	Ca+	4.06	26.4	47.2	1.32	67.5	0.48	9.3	11.4	0.36	23.3
LSL	P−	Ca−	3.84	21.6	37.3	1.05	72.9	0.53	8.1	10.7	0.38	27.9
Pooled SEM			0.114	3.49	7.12	0.117	2.47	0.015	0.58	1.04	0.017	2.45
*p*-values	Strain		0.187	0.485	0.273	0.990	0.848	0.155	0.222	0.590	0.128	0.373
	P		0.571	0.492	0.557	0.788	0.788	<0.001	0.308	0.887	<0.001	0.556
	Ca		0.004	0.027	0.014	<0.001	<0.001	<0.001	0.182	0.515	0.299	0.013
	Strain × P		0.696	0.555	0.360	0.236	0.228	0.712	0.051	0.220	0.289	0.351
	Strain × Ca		0.432	0.669	0.605	0.602	0.840	0.433	0.655	0.463	0.817	0.356
	P × Ca		0.071	0.691	0.417	0.513	0.835	0.311	0.077	0.817	0.578	0.167
	Strain × P × Ca		0.215	0.933	0.316	0.147	0.268	0.232	0.719	0.519	0.781	0.408

Data are given as LSmeans; *n* = 8–10 hens. ^1^ LB = Lohmann Brown-Classic. ^2^ LSL = Lohmann LSL-Classic.

**Table 8:** Effect of strain, P, and Ca on transcript copy numbers of Ca and P transporters in the ileum of laying hens aged 31 weeks. Copy numbers are displayed as log2 values.

Strain	Dietary P	Dietary Ca	ATP2B1	CALB1	NCX1	SLC20A1	SLC20A2	SLC34A2
LB ^1^	P+	Ca+	14.70	21.01	9.39	12.64	12.26	14.02
LB	P+	Ca−	14.99	21.29	9.16	13.08	12.34	14.26
LB	P−	Ca+	14.72	20.64	9.32	12.63	12.09	13.87
LB	P−	Ca−	14.80	21.05	9.06	13.18	12.23	14.16
LSL ^2^	P+	Ca+	14.30	21.05	9.47	11.90	11.90	13.26
LSL	P+	Ca−	14.56	21.39	9.64	12.05	12.01	13.83
LSL	P−	Ca+	14.49	21.47	9.56	12.44	12.24	13.78
LSL	P−	Ca−	14.61	21.45	9.53	12.44	12.10	13.74
Pooled SEM			0.252	0.227	0.252	0.306	0.131	0.229
*p*-values	Strain		0.077	0.081	0.083	<0.001	0.089	0.007
	P		0.898	0.827	0.779	0.189	0.596	0.760
	Ca		0.221	0.090	0.605	0.156	0.531	0.090
	Strain × P		0.488	0.067	0.834	0.278	0.027	0.270
	Strain × Ca		0.993	0.526	0.343	0.277	0.407	0.986
	P × Ca		0.568	0.670	0.740	0.954	0.553	0.364
	Strain × P × Ca		0.914	0.392	0.797	0.741	0.309	0.295

^1^ LB = Lohmann Brown-Classic. ^2^ LSL = Lohmann LSL-Classic. ATP2B1, ATPase plasma membrane Ca2+ transporting 1; CALB1, Ca binding protein 1; NCX1, sodium-Ca exchanger member 1; SLC20A1, solute carrier family 20 member 1; SLC20A2, solute carrier family 20 member 2; SLC34A2, solute carrier family 34 member 2.

**Table 9:** Effect of strain, P, and Ca on concentrations and contents of *myo*-inositol in the albumen, yolk, and egg (without shell) of laying hens aged 31 weeks.

			Albumen	Albumen	Yolk	Yolk	Egg
Strain	Dietary P	Dietary Ca	µmol/g	µmol	µmol/g	µmol	µmol
LB ^1^	P+	Ca+	0.54	18.8	0.79 ^b^	11.4 ^b^	30.2
LB	P+	Ca−	0.53	19.0	0.87 ^ab^	13.3 ^a^	32.3
LB	P−	Ca+	0.52	18.8	0.90 ^a^	13.6 ^a^	32.4
LB	P−	Ca−	0.51	19.2	0.87 ^ab^	12.9 ^ab^	32.0
LSL ^2^	P+	Ca+	0.47	17.2	0.84 ^ab^	13.5 ^a^	30.7
LSL	P+	Ca−	0.50	17.5	0.82 ^ab^	13.4 ^a^	30.9
LSL	P−	Ca+	0.51	18.4	0.79 ^b^	13.1 ^a^	31.5
LSL	P−	Ca−	0.50	18.8	0.85 ^ab^	14.3 ^a^	33.1
Pooled SEM			0.030	1.21	0.032	0.67	1.46
*p*-values	Strain		0.324	0.433	0.341	0.185	0.909
	P		0.976	0.367	0.293	0.207	0.179
	Ca		0.967	0.658	0.324	0.169	0.305
	Strain × P		0.222	0.431	0.142	0.470	0.783
	Strain × Ca		0.735	0.995	0.940	0.997	0.997
	P × Ca		0.555	0.917	0.763	0.454	0.773
	Strain × P × Ca		0.577	0.990	0.023	0.025	0.266

Data are given as LSmeans; *n* = 9–10 hens. ^1^ LB = Lohmann Brown-Classic. ^2^ LSL = Lohmann LSL-Classic. ^a–b^ Different superscript letters within a column indicate significant interaction effects among strains, dietary P, and dietary Ca (*p <* 0.05).

## Supplementary Materials

The following are available online at https://www.mdpi.com/2076-2615/10/10/1736/s1: Table S1: Primer sequences used for qRT-PCR, Table S2: Effect of strain, P level, and Ca level on titanium recovery in excreta and titanium concentration in different segments of the digestive tract of laying hens aged 31 weeks (g/kg).
