# Supplementary material for: Phytate Degradation, Transcellular Mineral Transporters, and Mineral Utilization by Two Strains of Laying Hens as Affected by Dietary Phosphorus and Calcium
Source: Animals (Basel). 2020 Sep 24;10(10):1736. doi: 10.3390/ani10101736 (PMC7598718; doi:10.3390/ani10101736)
Supplement: Supplementary file 1 [file animals-10-01736-s001.pdf]

Table S1. Primer sequences used for qRT-PCR

Primers	Melting temp (°C)	Sequence (5′-3′)	Amplicon length (bp)
**gga_ACTB_F1***	60	CCTCTTCCAGCCATCTTTCTT	254
**gga_ACTB_R1***	60	TAGAGCCTCCAATCCAGACA	
**gga_GAPDH_F1***	60	AGTCGGAGTCAACGGATTTG	251
**gga_GAPDH_R1***	60	CTGCCCATTTGATGTTGCTG	
**gga_ATP2B1_F1**	57	TTGCTGTTGTTGGGATTGAAG	179
**gga_ATP2B1_R1**	57	GCCCTCCAAACACAGAAAGTC	
**gga_CALB1_F1**	57	ATGCACTACTGAAGGATCTCTGTG	124
**gga_CALB1_R1**	57	GCCAGTTCTGCTCGGTAAAG	
**gga_NCX1_F1**	57	ACGGTGGACAAGCTCATTAAG	135
**gga_NCXI_R1**	57	CTTCTCCTCCCCACATTCATC	
**gga_SLC20A1_F1**	60	CTCTCGTCGTCTGGTTCTTTG	95
**gga_SLC20A1_R1**	60	CTTCTCCATCAGCGGACTTTC	
**gga_SLC20A2_F1**	60	TGCTGCTACCATTGCTATTAACG	161
**gga_SLC20A2_R1**	60	TTCTCTTCATCCAGGGGCATAC	
**gga_SLC34A2_F1**	60	CTGATCTTGCCATCGGTCTC	170
**gga_SLC34A2_R1**	60	TCCAGCCAGCCAAGTAAAAG	

*primers for amplicons of housekeeping genes; *ACTB* – beta-actin; *GAPDH* – glyceraldehyde 3-phosphate dehydrogenase; *ATP2B1* – ATPase plasma membrane Ca2+ transporting 1; *CALB1* – Ca binding protein 1; *NCX1* – sodium-Ca exchanger member 1; *SLC20A1* – solute carrier family 20 member 1; *SLC20A2* – solute carrier family 20 member 2; *SLC34A2* – solute carrier family 34 member 2

Table S2. Effect of strain, P level, and Ca level on titanium recovery in excreta and titanium concentration in different segments of the digestive tract of laying hens aged 31 weeks (g/kg).

Strain	Dietary P	Dietary Ca	Gizzard	Jejunum	Ileum	Recovery in excreta (%)
LB[1]	P+	Ca+	1.2	6.4	10.6	91.1
LB	P+	Ca-	1.0	6.1	11.4	88.8
LB	P-	Ca+	1.1	8.1	12.3	87.8
LB	P-	Ca-	1.0	7.0	11.4	93.6
LSL[2]	P+	Ca+	0.6	5.3	9.5	95.3
LSL	P+	Ca-	0.6	5.5	10.6	90.2
LSL	P-	Ca+	0.7	5.1	8.8	91.8
LSL	P-	Ca-	0.9	5.6	9.9	93.9
Pooled SEM			0.11	0.49	0.78	2.73
P-values	Strain		0.001	0.003	0.011	0.243
	P		0.196	0.020	0.961	0.807
	Ca		0.995	0.487	0.256	0.948
	Strain × P		0.091	0.010	0.166	0.858
	Strain × Ca		0.091	0.080	0.260	0.325
	P × Ca		0.144	0.677	0.416	0.025
	Strain × P × Ca		0.600	0.339	0.488	0.886

Data are given as LSmeans; *n* = 8-10 hens.

[1]LB = Lohmann Brown-Classic

[2]LSL = Lohmann LSL-Classic
